# Determination of the interaction between the receptor binding domain of 2019-nCoV spike protein, TMPRSS2, cathepsin B and cathepsin L, and glycosidic and aglycon forms of some flavonols

**DOI:** 10.3906/biy-2104-51

**Published:** 2021-08-30

**Authors:** Erman Salih İSTİFLİ, Arzuhan ŞIHOĞLU TEPE, Paulo A. NETZ, Cengiz SARIKÜRKCÜ, İbrahim Halil KILIÇ, Bektaş TEPE

**Affiliations:** 1 Cukurova University, Faculty of Science and Literature, Department of Biology, Adana Turkey; 2 Kilis 7 Aralık University, Vocational High School of Health Services, Department of Pharmacy Services, Kilis Turkey; 3 Theoretical Chemistry Group, Institute of Chemistry, Universidade Federal do Rio Grande do Sul, Porto Alegre Brazil; 4 Afyonkarahisar Health Sciences University, Faculty of Pharmacy, Department of Analytical Chemistry, Afyonkarahisar Turkey; 5 Gaziantep University, Faculty of Science and Literature, Department of Biology, Gaziantep Turkey; 6 Kilis 7 Aralik University, Faculty of Science and Literature, Department of Molecular Biology and Genetics, Kilis Turkey

**Keywords:** 2019-nCoV, spike glycoprotein, TMPRSS2, CatB, CatL, flavonol, molecular docking, molecular dynamics, MM/PBSA

## Abstract

The novel coronavirus (COVID-19, SARS-CoV-2) is a rapidly spreading disease with a high mortality. In this research, the interactions between specific flavonols and the 2019-nCoV receptor binding domain (RBD), transmembrane protease, serine 2 (TMPRSS2), and cathepsins (CatB and CatL) were analyzed. According to the relative binding capacity index (RBCI) calculated based on the free energy of binding and calculated inhibition constants, it was determined that robinin (ROB) and gossypetin (GOS) were the most effective flavonols on all targets. While the binding free energy of ROB with the spike glycoprotein RBD, TMPRSS2, CatB, and CatL were –5.02, –7.57, –10.10, and –6.11 kcal/mol, the values for GOS were –4.67, –5.24, –8.31, and –6.76, respectively. Furthermore, both compounds maintained their stability for at least 170 ns on respective targets in molecular dynamics simulations. The molecular mechanics Poisson–Boltzmann surface area (MM/PBSA) calculations also corroborated these data. Considering Lipinski’s rule of five, ROB and GOS exhibited 3 (MW>500, N or O>10, NH or OH>5), and 1 (NH or OH>5) violations, respectively. Neither ROB nor GOS showed AMES toxicity or hepatotoxicity. The LD50 of these compounds in rats were 2.482 and 2.527 mol/kg, respectively. Therefore, we conclude that these compounds could be considered as alternative therapeutic agents in the treatment of COVID-19. However, the possible inhibitory effects of these compounds on cytochromes (CYPs) should be verified by in vitro or in vivo tests and their adverse effects on cellular energy metabolism should be minimized by performing molecular modifications if necessary.

## 1. Introduction

Coronaviruses are positive sense RNA viruses with a diameter of 60–140 nm. As a result of electron microscopy studies, they were named as coronavirus since they carry spike proteins that cause a crown-like appearance on their surfaces (Richman et al., 2016). So far, four types of coronaviruses named as OC43, 229E, NL63, and HKU1 have been identified that circulate among humans. These pathogens usually cause mild respiratory infections in humans (Singhal, 2020).

In the past 20 years, two events have been recorded in which animal beta coronaviruses infected humans and caused serious consequences. In the first of these events, a beta coronavirus named as SARS-CoV passed from bats to humans via an intermediary host (palm civet cats) in the Guangdong province of China during the period of 2002–2003. SARS-CoV, which caused severe acute respiratory infection, affected 8422 people. However, the majority of the affected people lived in China and Hong Kong. The SARS-CoV epidemic caused 916 people to die (mortality rate 10.87%)(Chan-Yeung and Xu 2003). Approximately 10 years after this event, another beta coronavirus named as Middle East Respiratory Syndrome Coronavirus (MERS-CoV) appeared in Saudi Arabia. MERS-CoV has been transferred to humans using dromedary camels as the intermediate host. As a result of this epidemic, 2494 people were affected and 858 died (34.40% mortality rate) (Memish et al., 2020).

The third event in which another beta coronavirus caused an outbreak in humans occurred in Wuhan, China in late December 2019. This virus, named 2019-nCoV by the World Health Organization (WHO), has been identified as an infectious agent of respiratory tract similar to the SARS virus in humans. Then, the genome sequence of the virus was determined by the Shanghai Public Health Clinical Center and it was suggested that the pathogen was of bat origin (Chan et al., 2020). The cases were reported to originate from the Huanan Seafood Wholesale Market (Huang et al., 2020). It was understood that 2019-nCoV could be transferred among people, with the infection transmitted from a patient, who was being treated in a hospital in Wuhan city, to 15 healthcare professionals in close contact (Wang et al., 2020). As of May 26, 2021, 2019-nCoV reached 168,867,700 cases from all over the world, causing the death of 3,506,342 people.^1^Outbreak C (2020). Coronavirus Outbreak [online]. Website https://www.worldometers.info/coronavirus/ [Accessed 24 April 2020]


It is known that 2019-nCoV recruits the ACE2 receptor as the first gate in the process of entering the host cell. As a result of studies investigating the molecular interaction of the spike glycoprotein of the virus with the ACE2 receptor, it has been determined that leucine (455), phenylalanine (486), glutamine (493), serine (494), asparagine (501), and tyrosine (505) located in the receptor binding domain (RBD) of the spike protein play a primary role in the interaction (Zakaryan et al., 2017; Andersen et al., 2020). Following the binding of the RBD to the host cell receptor, the proteolytic cleavage of S protein at the S1/S2 interface and S2’ sites with the help of transmembrane protease, serine 2 (TMPRSS2), and/or cathepsins B/L (CatB/L) allows access to the host cellular cytosol (Simmons et al., 2005;Kawase et al., 2012; Zhou et al., 2015; Shirato et al., 2017; Shirato et al., 2018; Iwata-Yoshikawa et al., 2019; Cannalire et al., 2020). These first steps that SARS-CoV-2 utilize in its entry into the host cell are a unique cascade that can be targeted in reducing or completely abolishing the viral capacity of the virus. This type of blockage can only be achieved by simultaneous inhibition of spike, TMPRSS2, CatB, and CatL proteins. In a previous molecular modelling study on the ability of flavonoid molecules to block SARS-CoV-2 infection, such an approach has been shown to be rational (Istifli et al., 2020).

Many researchers have revealed that phytochemicals (especially flavonoids) are excellent compounds with strong antiviral effects on colds, flu, and other infectious diseases. Besides Madagascar, India has also decided to promote the use of plant-based phytochemicals to combat of COVID-19 infection (del Barrio et al., 2011; Vazquez-Calvo et al., 2017; Cataneo et al., 2019; Chen et al., 2019; Chen et al., 2019; Dai et al., 2019; LeCher et al., 2019; Mohd et al., 2019; Nagai et al., 2019; Parvez et al., 2019; Sochocka et al., 2019; Trujillo-Correa et al., 2019; Dwivedi et al., 2020; Ling et al., 2020; Lopes et al., 2020; Ritta et al., 2020; Tang et al., 2020). Flavonoids constitute a large group of polyphenols found in plants. They are examined under different groups in terms of their chemical structure (flavonols, flavones, flavanones, flavanols, anthocyanidins, isoflavones, dihydroflavonols, and chalcones).

Flavonols are one of the most common flavonoids in nature. Phytochemicals in this group are abundant in both aglycon and glycosidic form in the foods we consume frequently. It is known that flavonols, which are abundant in vegetables and fruits, are also noteworthy in wine, tea, grape, apple, and onion. The vast majority of flavonols are derived from the simplest built member, 3-hydroxyflavone. The best known flavonol is quercetin and is abundant in plants. Fisetin, morin, tamarixetin, isorhamnetin, myricetin, and kaempferol are other common flavonols. Among them, myricetin and kaempferol are common in many foods. Tamarixetin and isorhamnetin are structurally methylated metabolites of quercetin. After the consumption of this compound, the amounts of these phytochemicals increase in tissues or plasma. Studies show that daily intake of flavonol is 20–35 mg/day and quercetin and glycosides constitute more than half of this rate. As with many other phytochemicals, the bioavailability rate of flavonols depends on the presence of additional bound structures, such as oligosaccharide units that affect their solubility. Thus, the glycosidic forms of flavonols are more effective biological/pharmacological agents than aglycon forms (Dávalos et al., 2006; Perez-Vizcaino and Duarte 2010).

**Figure 1:**
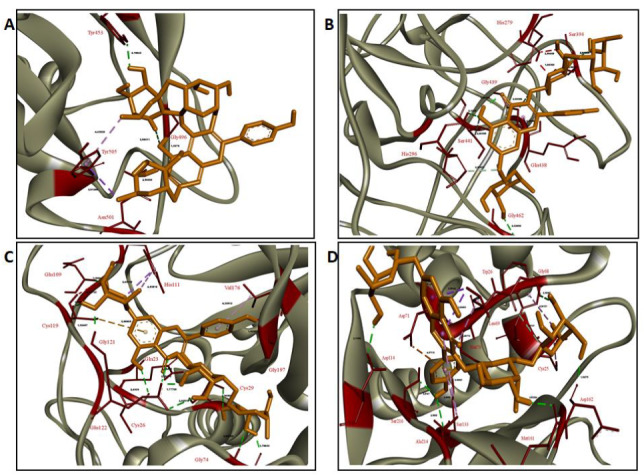
Chemical structures of the flavonols.

In this study, as mentioned above, the molecular interaction of certain flavonols (in the forms of aglycon and glycosidic) (Figure 1), which is an important subgroup of flavonoids, with the RBD of 2019-nCoV, TMPRSS2, CatB, and CatL was investigated by computer-based molecular docking and molecular dynamics analyses. Based on the binding free energy (kcal/mol) and calculated inhibition constant (mM) values obtained from docking analysis, ‘hit’ flavonols were determined by calculating relative binding capacity index (RBCI) and further molecular dynamics and MM/PBSA analyses were performed on these phytochemicals.

## 2. Materials and methods

### 2.1. Ligand preparation

In this study, the 23 ligands (3-hydroxyflavone, azaleatin, galangin, gossypetin, kaempferide, natsudaidain, pachypodol, rhamnazin, amurensin, fisetin, astragalin, azalein, morin, hyperoside, icariin, rhamnetin, myricitrin, kaempferitrin, quercitrin, robinin, troxerutin, spiraeoside, and xanthorhamnin) with their protein data bank (pdb) file formats were retrieved from PubChem.^2^https://pubchem.ncbi.nlm.nih.gov The geometry of the ligands were optimized using MMFF94 force field implemented in the Avogadro software.

### 2.2. Protein preparation using nanoscale molecular dynamics (NAMD)

Energy minimization of all receptor proteins (2019-nCoV ACE2-RBD, TMPRSS2, CatB, and CatL) were performed using NAMD according to the method given in the literature (Pedretti et al., 2004; Camacho et al., 2009; Remmert et al., 2012). Details were given in the supplementary file.

### 2.3. TMPRSS2 homology modeling

In line with the literature data regarding TMPRSS2 (Colovos and Yeates, 1993; Laskowski et al., 1993; Guex et al., 2009; Remmert et al., 2012; Studer et al., 2020), details on the process of generating the homology model were given in the supplementary file.

### 2.4. Molecular docking studies

Molecular docking studies were performed following the method given in the literature (Sanner, 1999; Greenspan et al., 2001; Wilson et al., 2005; Morris and Lim-Wilby 2008; Morris et al., 2009; Hardegger et al., 2011; Nasab et al., 2017; Andersen et al., 2020; Lan et al., 2020; Omotuyi et al., 2020; Wang et al., 2020; Woo et al., 2020; De Andrade et al., 2021) and details of the process were given in the supplementary file.

### 2.5. Calculation of the relative binding capacity index (RBCI)

Considering the activities of flavonols discussed in the present study against all four targets, RBCI analysis was carried out by following the method given in the literature in order to determine the “hit” compounds (Sharma, 1996; Istifli et al., 2020). Details of the applied RBCI method are given in the supplementary file.

### 2.6. Drug-likeness and ADMET prediction

The drug-likeness and ADMET profile of hit flavonols were determined according to the methods given in the literature (Delaney, 2004; Vistoli et al., 2008; Pires et al., 2015; Daina et al., 2017; Daina et al., 2019) and details were given in the supplementary file.

### 2.7. Molecular dynamics analyzes and molecular mechanics Poisson–Boltzmann surface area (MM/PBSA) calculations

Molecular dynamics and MM/PBSA analyzes were performed following the methods elsewhere (Parrinello and Rahman, 1981; Jorgensen et al., 1983; Hoover 1985; Darden et al., 1993; Essmann et al., 1995; Baker et al., 2001; Jakalian et al., 2002; Wang et al., 2004; Duan et al., 2009; O’Boyle et al., 2011; Homeyer and Gohlke 2012; Sousa da Silva and Vranken, 2012; Kumari et al., 2014; Abraham et al., 2015). Details of these molecular simulations were included in the supplementary file.

## 3. Results

The name of the flavonols, their PubChem CIDs, molecular weights, molecular formula, binding affinity (kcal/mol), and calculated inhibition constants (mM) along with their mean values and standard deviations (SD) were given in Table 1.

**Table 1:** PubChem CID, molecular weight, molecular formula, free energy of binding and calculated inhibition constant values of the compounds.

No	Compound	PubChem CID	Molecularweight (g/mol)	Molecularformula	Free energy of binding (kcal/mol)				Calculated inhibition constant (mM)			
					SpikeRBD	TMPRSS2	CatB	CatL	SpikeRBD	TMPRSS2	CatB	CatL
1	3-Hydroxyflavone	11349	238.24	C15H10O3	-5.61	-5.68	-6.96	-6.32	0.076	0.068	0.008	0.023
2	Azaleatin	5281604	316.26	C16H12O7	-4.64	-4.39	-7.34	-5.93	0.398	0.610	0.007	0.044
3	Fisetin	5281614	286.24	C15H10O6	-5.40	-5.13	-7.45	-6.29	0.109	0.172	0.003	0.024
4	Galangin	5281616	270.24	C15H10O5	-5.45	-4.03	-6.43	-6.02	0.100	0.702	0.019	0.038
5	Gossypetin	5280647	318.23	C15H10O8	-4.67	-5.24	-8.31	-6.76	0.380	0.144	0.0008	0.011
6	Kaempferide	5281666	300.26	C16H12O6	-5.38	-4.24	-6.58	-6.55	0.113	0.779	0.014	0.015
7	Morin	5281670	302.23	C15H10O7	-5.83	-4.74	-6.31	-6.18	0.053	0.335	0.023	0.029
8	Natsudaidain	3084605	418.40	C21H22O9	-5.27	-2.48	-7.25	-5.27	0.138	15.31	0.004	0.136
9	Pachypodol	5281677	344.30	C18H16O7	-5.80	-4.49	-7.21	-6.26	0.055	0.510	0.005	0.025
10	Rhamnazin	5320945	330.29	C17H14O7	-5.76	-4.56	-7.22	-5.96	0.059	0.451	0.005	0.043
11	Rhamnetin	5281691	316.26	C16H12O7	-4.94	-4.79	-7.49	-6.22	0.240	0.306	0.003	0.027
12	Amurensin	5318156	534.50	C26H30O12	-3.53	-0.99	-7.81	-6.16	2.61	188.46	0.002	0.030
13	Astragalin	5282102	448.40	C21H20O11	-6.35	-0.45	-8.51	-6.48	0.022	469.13	0.0005	0.017
14	Azalein	5321320	462.4 0	C22H22O11	-4.33	-1.42	-6.6	-7.05	0.675	90.76	0.014	0.006
15	Hyperoside	5281643	464.40	C21H20O12	-5.65	-1.98	-7.24	-5.93	0.071	35.49	0.005	0.044
16	Icariin	5318997	676.70	C33H40O15	-3.43	+9.01	-7.05	-6.79	3.07	nd1	0.006	0.010
17	Kaempferitrin	5486199	578.50	C27H30O14	-3.82	+1.85	-7.51	-7.49	1.58	nd1	0.003	0.003
18	Myricitrin	5281673	464.40	C21H20O12	-3.89	-0.89	-7.69	-6.80	1.40	224.1	0.002	0.010
19	Quercitrin	5280459	448.40	C21H20O11	-5.23	-1.74	-6.98	-6.96	0.145	0.531	0.007	0.007
20	Robinin	5281693	740.70	C33H40O19	-5.02	-7.57	-10.10	-6.11	0.209	0.0028	0.00005	0.033
21	Spiraeoside	5320844	464.40	C21H20O12	-3.19	-4.02	-6.39	-5.97	4.57	1.13	0.020	0.042
22	Troxerutin	5486699	742.70	C33H42O19	+0.44	+47.31	-4.06	-2.77	nd1	nd1	1.06	9.36
23	Xanthorhamnin	5351495	770.70	C34H42O20	-4.13	+18.67	-6.71	-5.99	0.941	nd1	0.012	0.040
	Mean				-4,65	0,35	-7,18	-6,19	0,773	57,17	0,05	0,44
	SD				1,41	11,65	1,07	0,88	1,200	122,89	0,22	1,95

nd: not determined (calculated inhibition constant value could not be determined because the binding energy of the molecule is positive)

### 3.1. Molecular interaction of flavonols with the RBD of the spike glycoprotein

Detailed data on the nonbonded interactions of flavonols with the 2019-nCoV RBD was given in Table S2 (see the supplementary file). According to the data in the table, the Van der Waals contacts were the leading interactions in the receptor-ligand interplay. Conventional and nonconventional H bonds were also found to be effective in these interactions. Considering the heatmap given in Figure S3, the flavonols that interacted most intensely with the RBD of the spike glycoprotein were kaempferide, natsudaidain, astragalin, kaempferitrin, spiraeoside, and xanthorhamnin. While rhamnazin, astragalin, icariin, myricitrin, quercitrin, and robinin (ROB) interacted extensively with catalytic residues of the RBD of the spike glycoprotein (Tyr505, Asn501, Ser494, Gln493, and Leu455), no molecular interactions were detected with Phe486, another active amino acid residue. According to the data in Table 1, the flavonol that had the strongest interaction with spike glycoprotein was astragalin. The binding affinity and calculated inhibition constant of this compound were determined to be –6.35 kcal/mol and 0.022 mM, respectively.

### 3.2. Molecular interaction of flavonols with TMPRSS2

The molecular interaction of flavonols with TMPRSS2 was given in Table S3 in the supplementary file. Similar to the interaction of flavonols with spike glycoprotein, Van der Waals contacts were found to dominate the interactions of flavonols with TMPRSS2. Classical H bonds were also detected in the flavonol-TMPRSS2 interaction. According to the heatmap given in Figure S4, flavonols exhibiting the most intense interaction with TMPRSS2 were 3-hydroxyflavone, azaleatin, fisetin, galangin, gossypetin (GOS), rhamnetin, amurensin, astragalin, quercitrin, and spiraeoside. While flavonols showed a significant interaction with the active amino acid residues of TMPRSS2, His296 and Ser441, only 3-hydroxyflavone was found to interact with another active amino acid, Asp345. Flavonols with binding free energy greater than -5.0 kcal/mol were 3-hydroxyflavone, fisetin, ROB, and GOS (Table 1). On the other hand, binding free energies of icariin, kaempferitrin, troxerutin and xanthorhamnin were found to be unfavorable (positive).

In order to explain in more detail, the molecular interactions of ROB and GOS with TMPRSS2, the binding mode of these two ligands was compared with Nafamostat, a proved experimental inhibitor of TMPRSS2 (PDB ID: 7MEQ). Nafamostat formed conventional hydrogen bonds with Asp435, Ser436, Gly439, and Gly464 residues of TMPRSS2, and also participated in the formation of carbon-hydrogen bonds with Gln438 and Gly472 residues. Nafamostat has additionally established Van der Waals contacts with residues Cys437, Asp440, Thr459, Trp461, Gly462, Cys465, Ala466, Arg470, and Pro471 (Figure S7). ROB has established conventional hydrogen bonds with His279, Gln317, Lys340, and Gly439 residues of TMPRSS2, as well as pi-donor hydrogen bonds and pi-pi T-shaped hydrophobic contacts with His296 residue. The ROB has additionally formed an alkyl bond with the Lys340 residue. In addition, van der Waals contacts formed with Val278, Val280, Cys281, Gly282, Cys297, Glu299, Tyr337, Thr341, Lys342, Trp384, Gly385, Thr393, Asp440, Ser441, and Trp461 residues played an important role for the snug fit of ROB into the catalytic pocket of TMPRSS2.

GOS formed conventional hydrogen bonds with Asn433, Asp435, Asp440, and Cys465 residues of TMPRSS2, and also participated in the formation of hydrophobic contacts with Cys437 (pi-alkyl), Cys465 (amide pi-stacked), and Ala466 (pi-sigma) residues. GOS has additionally established an electrostatic pi-cation interaction with Ala386. In addition, Van der Waals contacts formed with Gly259, Ile381, Gly385, Glu388, Glu389, Asn398, Ala400, Val434, Ser436, and Lys467 residues played an important role for the snug fit of GOS into the catalytic pocket of TMPRSS2. To summarize, ROB interacted with TMPRSS2, similar to Nafamostat, via residues Gly439 and Trp461. GOS, like Nafamostat, interacted with Asp435, Ser436, Cys437, Cys465, and Ala466 residues of TMPRSS2. The residue Asp440 was the amino acid commonly used by Nafamostat, ROB, and GOS in binding to TMPRSS2. Finally, ROB also formed chemical bonds with His296 and Ser441, which are the residues in the catalytic triad of TMPRSS2.

### 3.3. Molecular interaction of flavonols with CatB and CatL

Molecular interactions of flavonols with CatB and CatL were given in Tables S4 and S5 in the supplementary file. Van der Waals contacts and conventional H bonds are among the prominent nonbonded interactions in the molecular contacts of flavonols with cathepsins. While nonclassical H bonds, hydrophobic and electrostatic interactions stand out in the interaction of flavonols with CatB, mixed π/alkyl, electrostatic, lone pair/π-sulfur, Van der Waals interactions and classical H bonds were effective in interaction with CatL.

As can be seen from Figure S5 (supplementary file), where the flavonol-CatB interaction was visualized, flavonols interacted extensively with the active amino acid residues of CatB. It was determined that the majority of flavonols interacted extensively, especially with His111. The flavonols that interacted most with the active amino acid residues of CatB were as follows: 3-hydroxyflavone, azaleatin, fisetin, galangin, gossypetin, kaempferide, morin, natsudaidain, pachypodol, rhamnazin, rhamnetin, amurensin, astragalin, and kaempferitrin. Among the flavonols, the compound with the highest affinity for CatB was ROB with binding free energy of –10.10 kcal/mol and calculated inhibition constant value of 0.00005 mM.

Data on the interaction of flavonols with CatL was given in Figure S6 (supplementary file). According to data presented, flavonols interacted with the Cys25, Gly67, Gly68, Leu69, Met70, and Met161 active amino acid residues of CatL flavonols also interacted with Asp114, Ile115, and Lys117. The flavonol with the highest affinity for CatL was kaempferitrin. The free energy of binding and calculated inhibition constant of this compound were determined to be –7.49 kcal/mol and 0.003 mM, respectively.

### 3.4.Results of RBCI analyses (determination of ‘hit’ flavonols)

**Figure 2:**
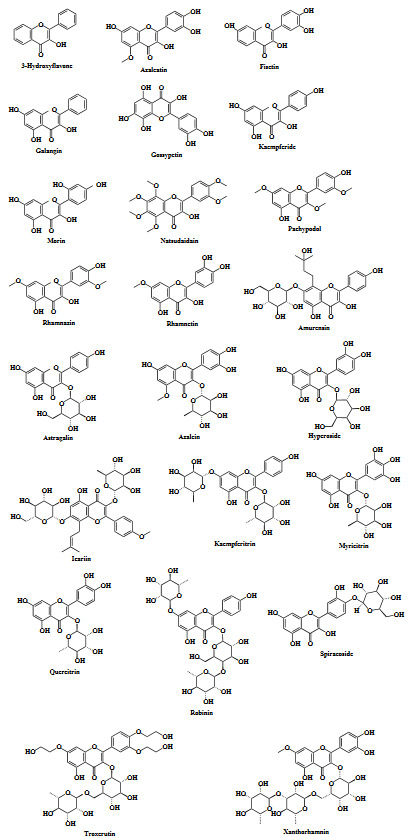
RBCI of the flavonols.

**Figure 3:**
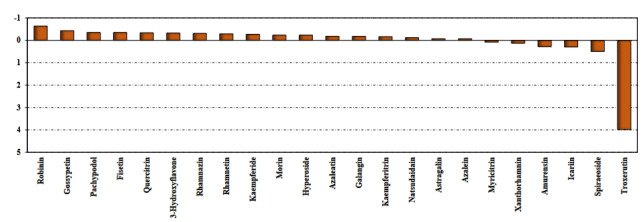
Top ranked conformations of robinin. (A- RBM of the spike glycoprotein of 2019-nCoV, BTMPRSS2, C- CatB, D- CatL).

**Figure 4:**
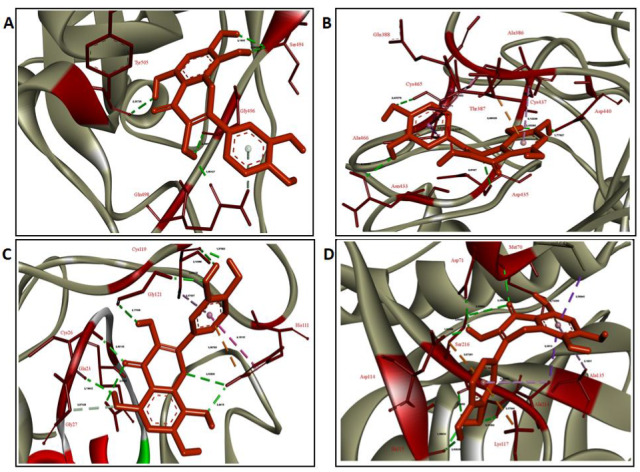
Top ranked conformations of gossypetin. (A- RBM of the spike glycoprotein of 2019-nCoV, B- TMPRSS2, C- CatB, D- CatL).

In this study, since the free energy of binding and calculated inhibition constants of the flavonols given in Table 1 against four different targets were calculated separately, RBCI analysis was applied to see how the affinity of flavonols on the respective targets were ranked when all targets were considered together. The ranking obtained using the RBCI analysis was given in Figure 2. The numerical values regarding this ranking were also presented in Table S1. As a result of the applied RBCI method, it was determined that the flavonols with the highest affinity to all target proteins were ROB and GOS. The RBCI coefficients of these compounds were determined as –0.64 and –0.43, respectively. Based on this finding, ROB and GOS were determined as ‘hit’ compounds. Due to page constraints, to be more reader friendly, instead of giving the top-ranked conformation of all flavonols, the top-ranked conformation for ROB and GOS is presented in Figures 3 and 4, respectively. Therefore, instead of performing molecular dynamics analysis of all compounds, the next part of the study was continued with ROB and GOS.

### 3.5. Drug-likeness and ADMET predictions of the flavonols

The drug-likeness properties of flavonols were demonstrated in Table 2. According to Table 2, 3-hydroxyflavone, azaleatin, fisetin, galangin, kaempferide, morin, natsudaidain, pachypodol, rhamnazin, and rhamnetin did not show any violation. However, the ‘hit’ flavonols, GOS and ROB, exhibited 1 and 3 violations, respectively (GOS: NH or OH> 5 and ROB: NH or OH> 5, N or O> 10, MW> 500).

**Table 2:** Drug-likeness properties of docked flavonols.

No	Compound	Number of rotatable bonds	TPSA1	Consensus Log P	Log S (ESOL2)	Drug-likeness(Lipinski’s rule of five)
1	3-Hydroxyflavone	1	50.44	2.84	-4.05	Yes; 0 violation
2	Azaleatin	2	120.36	1.41	-3.02	Yes; 0 violation
3	Fisetin	1	111.13	1.55	-3.35	Yes; 0 violation
4	Galangin	1	90.90	1.99	-3.46	Yes; 0 violation
5	Gossypetin	1	151.59	0.96	-3.40	Yes; 1 violation: NH or OH>5
6	Kaempferide	2	100.13	2.00	-3.51	Yes; 0 violation
7	Morin	1	131.36	1.20	-3.16	Yes; 0 violation
8	Natsudaidain	7	105.82	2.71	-4.17	Yes; 0 violation
9	Pachypodol	4	98.36	2.61	-4.46	Yes; 0 violation
10	Rhamnazin	3	109.36	2.02	-3.56	Yes; 0 violation
11	Rhamnetin	2	120.36	1.63	-3.36	Yes; 0 violation
12	Amurensin	7	210.51	0.44	-3.61	No; 3 violations: MW>500, N or O>10, NH or OH>5
13	Astragalin	4	190.28	-0.25	-3.18	No; 2 violations: N or O>10, NH or OH>5
14	Azalein	4	179.28	0.51	-3.20	No; 2 violations: N or O>10, NH or OH>5
15	Hyperoside	4	210.51	-0.25	-3.04	No; 2 violations: N or O>10, NH or OH>5
16	Icariin	9	238.20	0.84	-4.73	No; 3 violations: MW>500, N or O>10, NH or OH>5
17	Kaempferitrin	5	228.97	-0.46	-3.33	No; 3 violations: MW>500, N or O>10, NH or OH>5
18	Myricitrin	3	210.51	-0.23	-3.20	No; 2 violations: N or O>10, NH or OH>5
19	Quercitrin	3	190.28	0.16	-3.33	No; 2 violations: N or O>10, NH or OH>5
20	Robinin	8	308.12	-1.82	-3.33	No; 3 violations: MW>500, N or O>10, NH or OH>5
21	Spiraeoside	4	210.51	-0.19	-3.64	No; 2 violations: N or O>10, NH or OH>5
22	Troxerutin	15	297.12	-1.36	-2.79	No; 3 violations: MW>500, N or O>10, NH or OH>5
23	Xanthorhamnin	9	317.35	-1.90	-3.55	No; 3 violations: MW>500, N or O>10, NH or OH>5

^1^TPSA: Topological polar surface area (Å²) ^2^ESOL: Estimated aqueous solubility [(Insoluble < -10 < Poorly < -6 < Moderately < -4 < Soluble < -2 Very < 0 < Highly), according to Delaney, J.S. (2004)]. Data source: http://www.swissadme.ch/index.php#

ADMET of flavonols were shown in Table 3. Based on the data in Table 3, none of the flavonols, except 3-hydroxyflavone, were able to pass the blood-brain barrier (BBB). It was understood that not all flavonols were substrate of P-gp, except for amurensin, icariin, and ROB. Since almost half of flavonols have an inhibitory effect on CYPs, it was thought that they may adversely affect the energy metabolism of the cell. None of the flavonols showed neither AMES toxicity nor hepatotoxicity. The LD_50_ values of the ‘hit’ flavonols, ROB and GOS, in rats were determined as 2.482 and 2.527 mol/kg, respectively.

**Table 3:** ADMET profiles of flavonols.

No	Compound	BBB1 permeation1,*	P-gp substrate2,*	CYP inhibition3,*	AMES Toxicity4	Hepatotoxicity4	LD50 in rat(mol/kg)4
1	3-Hydroxyflavone	Yes	No	Yes (CYP1A2, CYP2C19, CYP2D6, CYP3A4)	Yes	No	1.991
2	Azaleatin	No	No	Yes (CYP1A2, CYP2D6, CYP3A4)	No	No	2.393
3	Fisetin	No	No	Yes (CYP1A2, CYP2D6, CYP3A4)	No	No	2.465
4	Galangin	No	No	Yes (CYP1A2, CYP2D6, CYP3A4)	No	No	2.450
5	Gossypetin	No	No	Yes (CYP1A2, CYP2D6, CYP3A4)	No	No	2.527
6	Kaempferide	No	No	Yes (CYP1A2, CYP2D6, CYP3A4)	No	No	2.338
7	Morin	No	No	Yes (CYP1A2, CYP2D6, CYP3A4)	No	No	2.413
8	Natsudaidain	No	No	Yes (CYP2C19, CYP3A4)	No	No	2.379
9	Pachypodol	No	No	Yes (CYP1A2, CYP2C9, CYP2D6, CYP3A4)	No	No	2.212
10	Rhamnazin	No	No	Yes (CYP1A2, CYP2C9, CYP2D6, CYP3A4)	No	No	2.241
11	Rhamnetin	No	No	Yes (CYP1A2, CYP2D6, CYP3A4)	No	No	2.453
12	Amurensin	No	Yes	No	No	No	2.634
13	Astragalin	No	No	No	No	No	2.546
14	Azalein	No	No	Yes (CYP3A4)	No	No	2.537
15	Hyperoside	No	No	No	No	No	2.541
16	Icariin	No	Yes	No	No	No	2.631
17	Kaempferitrin	No	Yes	No	No	No	2.587
18	Myricitrin	No	No	No	No	No	2.537
19	Quercitrin	No	No	No	No	No	2.586
20	Robinin	No	Yes	No	No	No	2.482
21	Spiraeoside	No	Yes	No	No	No	2.559
22	Troxerutin	No	Yes	No	No	No	2.476
23	Xanthorhamnin	No	Yes	No	No	No	2.477

^1^BBB: Blood Brain Barrier ^2^P-gp: P-glycoprotein substrate ^3^CYP: Cytochrome P ^4^http://biosig.unimelb.edu.au/pkcsm/prediction ^5^https://www.swissadme.ch

### 3.6. Molecular dynamic analyses and binding free energy (MM/PBSA) calculations of ‘hit’ flavonoids

**Figure 5:**
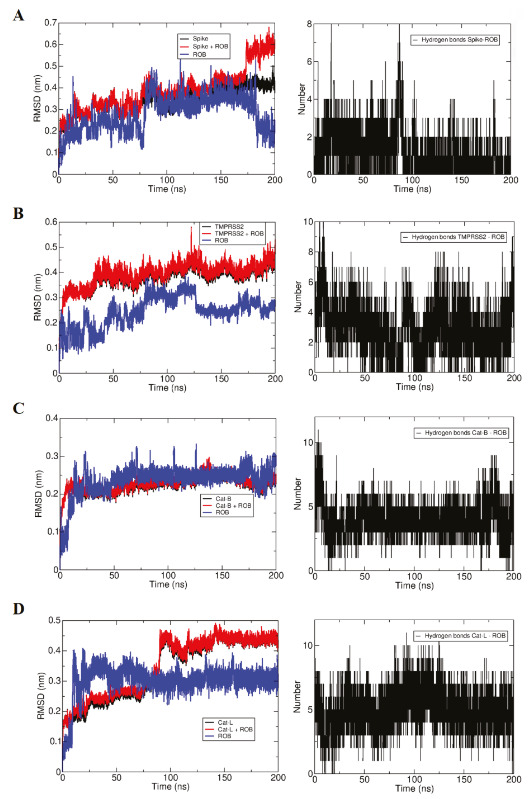
Time-dependent evolution of the structure and interactions of the robinin (ROB) molecule with target proteins in molecular dynamics analysis. A- RBD-ROB complex, B- TMPRSS2-ROB complex, C-CatB-ROB complex, D-CatL-ROB complex. On the left, the RMSD of the atomic positions of the receptor (black), ligand (blue), and the ligand + receptor (red) complex is given, while the right of the figure shows the number of hydrogen bonds between the ligand and the receptor.

The ROB molecule, initially interacted with the residues Tyr453, Tyr489, Phe490, Tyr495, Gly496, Asn501, and Tyr505 of spike’s RBM (Figure 5A). It remained interacting with the same residues until 77 ns, when the interaction with residues Tyr495, Gly496, and Tyr505 was lost and replaced with the interaction with the residues Phe456, Gly485, and eventually with Ala475. At 170 ns, a rearrangement took place and the ROB molecule interacted with the side loop (residues 475 until 479), remaining in this configuration until the end of the simulation. By the analysis of the trajectory and number of hydrogen bonds we can conclude that the interaction was moderately strong (Figure 5A).

The ROB molecule started interacting with the residues His279, Glu389, Val280, Lys390, Gly391, Lys392, Thr393, Gln438, Cys437, Gly439, Ser441, Asp440, and Ser463 of TMPRSS2 (Figure 5B). At 40 ns it started to interact also with Ser318 and Met320. At about 65 ns the ROB molecule underwent a conformational change and lost the contacts with the loop of the residues 389–393, but started to interact with His296, Lys340, Thr341, Lys342, Trp461, and Gly462. This conformation remained stable until the end of the simulation period. Analyzing the trajectory and the evolution of the hydrogen bond pattern let us to conclude that the interaction was strong (Figure 5B).

The ROB molecule started interacting with several residues of CatB: Ser25, Gln23, Gly27, Cys26, Cys29, Gly73, Gly74, Glu109, His111, His110, Val112, Pro118, Cys119, Gly121, Thr120, Glu122, Val176, Gly197, and Gly198 (Figure 5C). The interaction was very strong (with several hydrogen bonds) and, besides some rearrangement of external loops in CatB, the complex remained very stable. In the end of the simulation the interaction pattern is essentially the same, only the interactions with Gly121 and Glu122 were lost. By the analysis of the trajectory and the evolution of the hydrogen bond pattern we can conclude that the interaction was very strong (Figure 5C).

The ROB molecule started interacting with several residues CatL: Cys25, Trp26, Gly67, Gly68, Leu69, Met70, Asp71, Ser133, Val134, Asp160, Met161, Asp162, His163, Gly164, Ala214, and Ser216 (Figure 5D). At about 20 ns the ROB molecule suffered a reorientation and started to interact with Glu63, Asn66 and Gly159, losing the interactions with the residues 134 and 162–164. At about 85 ns the Cat-L molecule underwent a conformational transition in an outer loop with a short helix, not interacting with the ROB molecule. The pattern of interactions is essentially maintained throughout the simulation. In this case, again, by the analysis of the trajectory and the evolution of the hydrogen bond pattern we can conclude that the interaction was very strong (Figure 5D).

**Figure 6:**
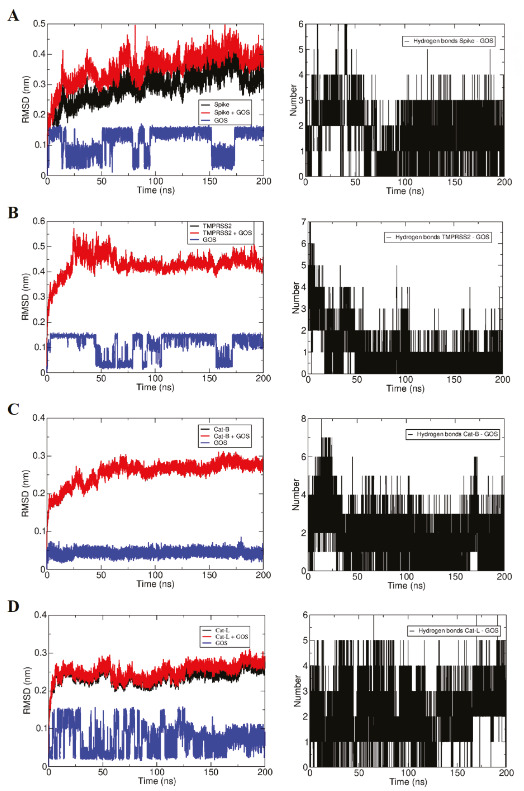
Time-dependent evolution of the structure and interactions of the gossypetin (GOS) molecule with target proteins in molecular dynamics analysis. A- RBD-GOS complex, B- TMPRSS2-GOS complex, C-CatB-GOS complex, D-CatL-GOS complex. On the left, the RMSD of the atomic positions of the receptor (black), ligand (blue), and the ligand + receptor (red) complex is given, while the right of the figure shows the number of hydrogen bonds between the ligand and the receptor.

The interaction of GOS, our second top-ranked ligand, with spike’s RBM is shown in Figure 6A. The GOS molecule started interacting with the residues Ser494, Tyr495, Gly496, Phe497, Gln498, and Tyr505. In a few nanoseconds, it migrated to the opposite edge of spike’s RBM, interacting with Glu484, Gly485, Phe486, Asn487, Cys488, and Tyr489, where it remains until 80 ns, interacting eventually with Gln493 and Thr470. It detached briefly at 81 ns, returns to interact with the RBM and starting at 90 ns, until the end of the simulation, it interacted with the outer edge of the loop containing the residues 439 until 445, as well as with Pro499 and Thr500. The molecule probably migrated from a region with weak interactions to a region with moderate interactions (Figure 6A).

The GOS molecule started interacting with the residues Ser382, Ala386, Thr387, Glu388, Glu389, Ala399, Asn433, Val434, Asp435, Ser436, Cys437, Cys465, and Ala466 of TMPRSS2 (Figure 6B). Suffering only some small rearrangements, the molecule remained interacting essentially in the same place during all the simulation, losing only the interactions with Glu388, Glu389, and Asn433. Despite the low number of hydrogen bonds, considering the residence of the ligand in the protein pocket, the interaction is expected to be moderately strong to strong (Figure 6B).

The GOS molecule initially formed interactions with the residues Gln23, Gly24, Ser25, Cys26, Gly27, His110, His111, Cys119, Thr120, Gly121, Glu122, Leu181, and Gly197 of CatB (Figure 6C). It remained essentially in the same region in the protein, making eventually additional interactions with the residues Cys29 and Met196 and in the end of the simulation also with Trp221 and Asn222. Even considering that the hydrogen bond interactions are not much high in number, the high residence time of the ligand in the protein pocket let us conclude that the interaction is expected to be strong (Figure 6C).

The GOS molecule started interacting with the residues Gly68, Leu69, Met70, Asp71, Asp114, Lys117, Ala135, Asp160, Met161, Asp162, His163, Ala214, Ala215, and Ser216 of CatL (Figure 6D). It remained in essentially the same position, but suffering some structural rearrangement, eventually interacting with the residue TRP26. In the end of the simulation, the contacts with the residues Asp114, Lys117, Ala214, Ala215, and Ser216 were lost and new contacts were made with Asn66 and Gly67. Even considering that the hydrogen bond interactions are not much high in number, the high residence time of the ligand in the protein pocket let us conclude that the interaction is expected to be strong (Figure 6D).

The RMSD of the ligands show that their structure shave converged in all simulations and remained stable, apart from some structural fluctuations related to torsional conformational transitions, yielding multiple similar conformations (in the case of ROB) and two conformations (in the case of GOS). The conformational transitions of the ligands, however, do not disturb the strong interactions with the receptors (Figures 5–6).

In the present, the binding free energy of the complexes formed by ROB and GOS with four different receptors was calculated by the MM/PBSA method. Both MM/PBSA and MM/GBSA are computational methods used to estimate the free energy of binding of small molecules to receptors (proteins, nucleic acids or other macromolecules) (Genheden and Ryde, 2015). In both methods the free energy of a system is calculated as a sum of contributions (bond, angle, dihedral, electrostatic, van der Waals, polar, and nonpolar solvation terms and entropy estimation)(Kollman et al., 2000). The binding free energy is calculated as a difference between the calculated free energies of complex (protein + ligand) and the free energies of the isolated protein and ligand, sampled over a large number of configurations, usually generated using molecular dynamics simulations. In the MM/PBSA method the Poisson–Boltzmann equation is used to calculate the polar solvation free energy contribution, whereas in the MM/GBSA method the (more simplified but faster) generalized Born model is employed for this purpose. Both methods yield similar results and their merits are discussed in the recent literature (Genheden and Ryde 2015; Sun et al., 2018). Being fully compatible with GROMACS as an additional program with GROMACS-like syntax, we employed the g_mmpbsa program which applies MM/PBSA method to molecular dynamics trajectories (Kumari et al., 2014).

In our study, the results obtained from docking and molecular dynamic analysis and the binding free energy values calculated by the MM/PBSA (Table 4) method corroborate each other. As evidenced by the negative binding free energies, ROB had favorable interactions with all other receptors. The ROB molecule made its strongest interaction with the CatB, while the weakest interaction was made with the TMPRSS2. These results are in agreement with the time-dependent evolution of the number of hydrogen bonds and qualitative analysis of simulations. Likewise, for GOS, MM/PBSA, docking, and molecular dynamics results support each other. The interactions of GOS with all receptors are favorable. While GOS performed its strongest interaction with CatB, the weakest interaction was found for GOS with spike glycoprotein. These results are consistent with the time-dependent evolution of the number of hydrogen bonds and qualitative analysis of the simulations. It is noteworthy that the interaction between ROB and CatB could be described as very strong by all methods: docking scores, MM/PBSA free energy calculations and also by the qualitative analysis of the molecular dynamics simulations.

**Table 4:** Free energies of binding and their components, calculated using MM/PBSA, for the interaction between robinin, gossypetin and the receptors A- Spike, B- TMPRSS2, C- CatB and D- CatL.

Ligand	Protein	Van der Waals Energy(kj/mol)	Electrostatic energy(kj/mol)	Polar solvation energy(kj/mol)	SASA energy(kj/mol)	Binding energy(kj/mol)
Robinin	Spike	-133.914 +/- 1.770 kJ/mol	-29.898 +/- 1.482 kJ/mol	118.541 +/- 3.002 kJ/mol	-14.707 +/- 0.253 kJ/mol	-59.879 +/- 1.585 kJ/mol
	TMPRSS2	-203.769 +/- 2.168 kJ/mol	-74.785 +/- 1.941kJ/mol	243.174 +/- 3.185 kJ/mol	-22.268 +/- 0.138 kJ/mol	-57.828 +/- 2.318 kJ/mol
	CatB	-231.051 +/- 1.660 kJ/mol	-66.831 +/- 1.873 kJ/mol	177.507 +/- 2.599 kJ/mol	-23.652 +/- 0.125 kJ/mol	-144.115 +/- 1.317 kJ/mol
	CatL	-179.982 +/- 2.207 kJ/mol	-148.681 +/- 3.039 kJ/mol	252.426 +/- 4.883 kJ/mol	-22.524 +/- 0.215 kJ/mol	-98.687 +/- 1.634 kJ/mol
Gossypetin	Spike	-70.987 +/- 1.297 kJ/mol	-47.116 +/- 2.691 kJ/mol	80.120 +/- 2.538 kJ/mol	-8.853 +/- 0.083 kJ/mol	-46.843 +/- 1.127 kJ/mol
	TMPRSS2	-150.764 +/- 1.223 kJ/mol	-34.114 +/- 2.083 kJ/mol	117.266 +/- 1.747 kJ/mol	-13.473 +/- 0.097 kJ/mol	-81.049 +/- 1.154 kJ/mol
	CatB	-161.606 +/- 0.711 kJ/mol	-24.490 +/- 1.229 kJ/mol	89.835 +/- 1.166 kJ/mol	-14.946 +/- 0.058 kJ/mol	-111.257 +/- 0.732 kJ/mol
	CatL	-122.410 +/- 1.236 kJ/mol	-60.958 +/- 2.313 kJ/mol	101.533 +/- 2.105 kJ/mol	-12.594 +/- 0.061 kJ/mol	-94.330 +/- 1.046 kJ/mol

## 4. Discussion

According to the literature records, there is no report on the interactions of GOS, natsudaidain, kaempferide, amurensin, azalein, icariin, spiraeoside, and xanthorhamnin with the target proteins in this study. On the other hand, there are studies that examine the inhibitory potentials of 3-hydroxyflavone (Batool et al., 2020), fisetin (Arora et al., 2020; Oladele et al., 2020), astragalin (Arora et al., 2020), morine (Laskar et al., 2020), galangin (Hashem, 2020), pachypodol (Ebada et al., 2020), rhamnazin (Swargiary et al., 2020), rhamnetin (Fischer et al., 2020; Kousar et al., 2020), hyperoside (Cherrak et al., 2020; De Jesús-González et al., 2020; Hu et al., 2020), astragalin (Hu et al., 2020), myricitrin (Abd El-Mordy et al., 2020; Joshi et al., 2020), quercitrin (Arora et al., 2020; Patel et al., 2020), and troxerutin (Kandeel et al., 2020) on several proteins of SARS-CoV-2. However, there have been no studies observed investigating the inhibitory effect of the above-mentioned phytochemicals on spike glycoprotein, TMPRSS2, CatB, and CatL simultaneously.

According to the literature records, there is one report examining the interaction of only 3-hydroxyflavone, among flavonols examined in the present study, with TMPRSS2 (Puttaswamy et al., 2020). According to this study, quercetin 3,5-diglucoside (–9.6 kcal/mol), myricetin 3-rutinoside (–9.4 kcal/mol), rutin (–9.3 kcal/mol), kaempferol (–9.2 kcal/mol), myricetin 3-rhamnoside (–9.2 kcal/mol), and robinetin 3-rutinoside (–9.6 kcal/mol) originating from the 3-hydroxyfavone chemical structure exhibited highly favorable binding free energies against TMPRSS2. These values were higher than the value (–5.68 kcal/mol) presented in the current study. It was thought that this difference may be due to minor differences in the glycosidic structures of the flavonols in question.

The flavonols examined in the present study were also subjected to literature research in terms of their interactions with spike glycoprotein of SARS-CoV-2. It was determined that the interactions of flavonols except fiset in (Jain et al., 2021; Vijayakumar et al., 2020), galang in (Jain et al., 2021), morin (Jain et al., 2021), astragalin (Adejoro et al., 2020; Hiremath et al., 2021), kaempferitrin (Arokiyaraj et al., 2020), quercitrin (Teli et al., 2020; Hiremath et al., 2021), and troxerutin (Somadi and Sivan, 2020) with spike glycoprotein were not analyzed. It is of course impossible to discuss here all of the data presented in these reports. In general, however, the binding free energy data obtained for flavonols given above appear to be consistent with those presented in the current study. It was thought that the negligible differences between the literature data and the existing data may be due to the use of different docking programs for in silico analysis.

On the other hand, the ROB molecule extracted from *Platycodi radix*, the root of the *Platycodon grandiflorum* plant, has been reported to bind to the 3CL pro enzyme of SARS-CoV-2 and to inhibit its proteolytic activity (Leung et al., 2020). In addition, in a different study carried out using the molecular docking method, gossypetin-3’-O-glucoside, a GOS derivative, has been reported to show highly favorable inhibitory potential (ΔG: –11.93 kcal/mol) by binding to Cys145 and His41 residues in the catalytic center of 3CLpro (Giguet-Valard et al., 2020). We are in the opinion that the reason behind the high affinity of ROB and GOS for the spike, TMPRSS2, CatB, and CatL enzymes in our computational study is that: hydrogen (H) bonds formed between the H atoms attached to -OH groups of ROB and GOS and the electron pairs on heteroatoms (such as N, O) of 4 different enzymes increase the stability of the receptor-ligand interactions. In a similar mechanism, the H-bonds formed between the electron pairs on the C=O (carbonyl) or oxygen atom (–O–) of ROB and GOS and the H atoms bound to the heteroatoms (such as N, O) of these 4 different enzymes also favor the stability of the receptor-ligand interactions.

As can be seen from the sections above, ROB and GOS were announced as ‘hit’ flavonols. No other computational study has been found investigating the inhibition potential of the ROB on a different virus other than SARS-CoV-2. However, there are some reports that GOS has antiviral activity on chikungunya virus (CHIKV), dengue virus (DENV), and Ebola virus (EBOV)(Raj and Varadwaj, 2016; Keramagi and Skariyachan, 2018). In a study by Keramagi and Skariyachan (2018), it was reported that GOS exhibited the most negative binding energy (kcal/mol) and maximum stabilizing interactions on CHIKV and DENV targets. In another study by Raj and Varadwaj (2016), it was reported that GOS exhibited highly significant docking scores on EBOV receptor proteins. These literature findings indicate that GOS has a high antiviral activity potential.

## 5. Conclusion

According to the results obtained from this study, ROB and GOS showed promising activities on the RBD of the spike glycoprotein of 2019-nCoV, TMPRSS2, and cathepsins. The employed methods (molecular docking, MM/PBSA free energy estimates, and qualitative MD analysis) are in close agreement in almost all cases. In particular, a remarkable strong interaction was found between ROB and CatB. Therefore, it has been concluded that these molecules can be considered as an alternative approach in the treatment of COVID-19 disease. Although these molecules showed neither AMES toxicity nor hepatotoxicity, it was thought-provoking that GOS has an inhibitory effect on CYPs and that ROB is a substrate of P-gp. However, according to SwissADME database, GOS still meets the criteria for drug likeness and, more importantly, lead likeness. Moreover, our other hit ligand, ROB, has been proven to be used as a reliable antidiabetic agent in an in vivo study, and as an antiinflammatory and antiarthritic drug in a different in vivo study (Srivastava et al., 2017; Tsiklauri et al., 2021). It was concluded that further in vitro and/or in vivo tests should be performed in order to reveal the ultimate toxicity of the molecules in question and their effects on cellular energy metabolism, and if necessary, the observed side effects should be minimized by molecular modifications. Thus, the theoretical data could hopefully be confirmed experimentally.
